# The Trials of a Young Doctor

**Published:** 1897-09

**Authors:** Jno. W. Kenney

**Affiliations:** San Antonio, Tex.


For the Texas Medical Journal.

### THE TRIALS OF A YOUNG DOCTOR.

BY JNO. W. KENNEY, M. D., SAN ANTONIO, TEX.

THE science of medicine has many truly fascinating charms; to the young man fresh from college with the finale of an Art's course fresh in his mind it is especially interesting. It presents to his vivid imagination a rare chance to do great good for suffering man—to make friendship ties that last beyond the grave—to furnish fields for fruitful study, and he sees spread out before him an aggregation of sciences united as one, in which to gain fame, and be known as a benefactor to this world.

Were this all his imagination tells him, it would be well. It is not all, however. The imagination too often proves a poor place to draw on for facts. We find this learned novice treading paths in Elysian fields, as it were, with his imagination for both foundation and guide. He pictures himself a self-constituted medical Moses in his community, and with this air indelibly impressed upon his countenance, already besmirched by an assumed dignity, and possibly an attempt at whisker-growing, he fancies that he possesses a coupe and team to carry him from door to door as he answers the summons of his clientelle.

If his imagination be of an average brand he will see the patrons of his professional brothers flocking to his office to receive the glad tiding—health—from his hand. There is a picture in his imagination, of a home, and a sanitarium of great magnitude and splendor, and a wife to share his joys, for of sorrow he will be free.

The first trial that the young doctor will meet will be that of disappointment. His friends shun him as though his learning were dangerous, and are the last to seek his professional advice. The patrons of older physicians prefer to continue their membership in the clientelle of their old family doctor. The coupe and the wherewith to carry it over the smooth roads prove a myth. The home and sanitarium do not materialize. He is more often annoyed by peddlers than patients while in his office. He changes his mind about wanting a wife to share his joys, and working upon the hypothesis that "misery loves company," he too often secures one to share his sorrows. He awakens to the painful fact that most of his patients engage him when some other cannot be found. He finds that even in these cases that respect which he much desires is lacking. I cannot better tell of the reception meted out to young doctors when answering emergency calls than by giving some of the experiences of a young friend of mine—in fact a very dear friend and associate. Early one morning he was called to see a dying child. As he entered the house his eyes fell upon some half dozen or more women, and among them was one, bolder than the rest, who informed my young friend that "she had never heard of him before." Of course this was humiliating to the young doctor, but he informed the lady that there were lots of great men in this world that she had never heard of; and proceeded to try and prevent the sick youth from dying a natural death.

On another occasion the young follower of Æsculapius, while treating a little girl for some slight ailment, ventured to ask the mother how she came to send for *him*. The mother replied that she had heard Mrs. —— speak very favorably about him, and that she thought he was forty or fifty years old, and then, to explain matters more fully, she added: "I thought you was somebody, and I like to have dropped dead when I first saw you." Again, when this same young doctor presented his bill to a representative citizen of this place for some profes sional service that he had by chance rendered, he was informed that he charged more than a good doctor.

It seems that there is no end to the insulting epithets that kind friends are wont to give him. He leaves the fields of his imagination and the cruel realism of his first few ventures and passes on to wishful pastures. He wishes for at least a share of this world's goods—for kind treatment by laymen and the members of his fraternity—for a sufficient number of folks to engage his services to prevent him forgetting all the knowledge gained while at school.

In this anxious mood many times disappointment reigns; the brotherly love that we read and hear about, but, sorry to say, seldom see among medical men, will be tested and found wanting in many instances.

Some fair patient will become skeptical about his ability as a diagnostician, and suggest a consultation with some older physician. This the young man is pleased to do; in fact, he is made glad by an opportunity to meet his more experienced brother at the bedside. He requests the physician of his patient's choice to meet him in the sick chamber. They meet and it is then that the novice receives his first lesson in the artifices of a practitioner of medicine. He may realize that this confidence has been misplaced—that instead of a friend he is consulting with a foe. This is quickly impressed upon his mind the moment the egotistic shining medical light enters the room, for straightway his conduct may be thus: Seats himself at the bedside of the patient; grasps her hand and squeezes it as though there was deep seated in his heart a love long drawn out for her welfare—smoothes back her disheveled tresses, and leaves not a ringlet misplaced; tells her that it is too bad that she has had to suffer so much, and that he will treat her as he would his mother or sister. While this is going on the young doctor stands by like the balance of the furniture in the room, with his anger more than bitter as he gradually allows himself to see that his consultant is making a desperate effort to gain favor in the patient's eyes. About this time the consulting physician may address a few remarks to the physician in charge, to learn of his manner of examining the case, and the treatment that had been given that he may more effectually instill his own greatness into the patient by entering upon a minute examination on an entirely different line to that followed by the novice, and likewise suggest a radical change of treatment. As a capstone to this already ludicrous spectacle, he winds up his consultation by delivering a clinical lecture upon the case in question and allied conditions—telling of the great number of similar cases he has treated during his professional career, and of the remarkably successful termination of each case.

Young physicians are wiser as a rule after passing through an ordeal like the one above described. They become more careful in their association with other physicians.

However, it is not consultation, as a rule, that troubles his restless brain. The lack of them more often proves the irritant. The time hangs heavily on his hands. His office hours are long and his profits small. His better reason tells him to bury his mind in books, but this proves a difficult task. In this frame of mind the young man may dwell for a considerable time. Seventy per cent. throw aside their profession and embark in more lucrative employment. Of the remaining thirty per cent. only ten ever amount to anything in the medical world. Thus we find the realism of medicine in marked contrast to the views we gain at the beginning. It is not all gloom that overshadows one engaged in the practice, for there is a bright side that is good. However, it is the trials that I am to tell of, and with the few that I have related my tale is told.

